# Supplementary material for: A-to-I mRNA Editing in a Ferric Siderophore Receptor Improves Competition for Iron in Xanthomonas oryzae pv. oryzicola
Source: Microbiol Spectr. 2021 Oct 27;9(2):e01571-21. doi: 10.1128/Spectrum.01571-21 (PMC8549721; doi:10.1128/Spectrum.01571-21)
Supplement: SUPPLEMENTAL FILE 1 — Supplemental material. Download SPECTRUM01571-21_Supp_1_seq10.pdf, PDF file, 0.8 MB [file spectrum01571-21_supp_1_seq10.pdf]

Appendixes

Figure S1. Normalized expression levels of selected chemotaxis pathway genes (*xoc_2278*, *xoc_2280*, xoc_2289, xoc_2291, and xoc_2297) and *xfeA* in *Xoc* BLS256. Wild-type BL256 was cultivated in NB, NB+50 μM 2,2'-dipyridyl (DP, iron chelator), and NB+100 μM $FeCl_3$. Gene expression levels were calculated relative to *rpoD* using the ΔΔCT method, where CT is the threshold cycle. Three independent biological replicates were carried out in this study.

Figure S2. Prediction of the mutation site in *Xoc* T408A. RNA secondary structure analysis (http://rna.tbi.univie.ac.at/) showed that the edited site was embedded within a loop (see arrow). Color is used to show base-pair probabilities.

Figure S3. Diagram of capillary chemotaxis assay.

Figure S4. Verification of *xfeA* T408A A-to-I editing site by Sanger sequencing using gDNA as the template. Chromatograms show editing in *Xoc* BL256 (wild-type, WT) grown in NB, NB+50 μM DP, NB+100 μM DP, NB+150 μM DP, and NB+100 μM $FeCl_3$. The *Xoc ΔtadA* and $T408^{silent}$ mutants were grown in NB and included for comparison.

Figure S5. Predicted secondary structure of XfeA based on analysis with the Phyre2 web site. Structure of XfeA in *Xoc* (a) $T408^{silent}$ (no editing) and (b) the T408A mutant (with editing). The latter mutant contains a truncation in the β-strand (red arrow and rectangle).

Figure S6. Homology modeling of XfeA showing the predicted site of ferrienterobactin binding. The model was constructed with AutoDock Vina.

Table S1. Strains and plasmids used in this study.

Table S2. Primers used in this study.

Table S3. Differentially regulated chemotaxis genes in *Xoc* T408A and T408^{silent} identified by RNA-seq.

Table S4. A-to-I RNA editing in *Xoc* T408A strain and T408^{silent} strain.

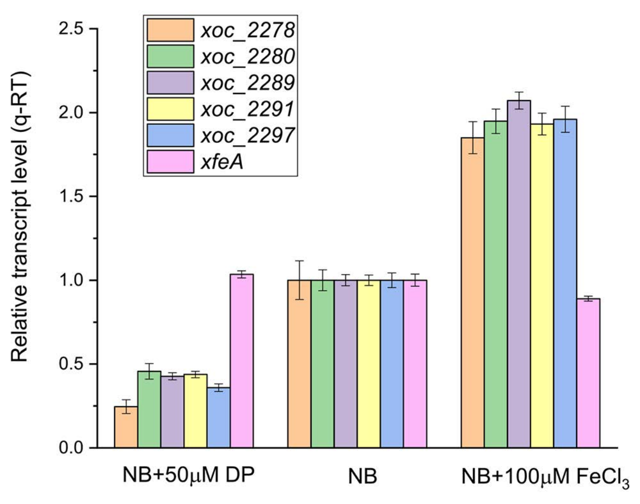

xoc_2278
xoc_2280
xoc_2289
xoc_2291
xoc_2297
xfeA
Relative transcript level (q-RT)
2.5
2.0
1.5
1.0
0.5
0.0
NB+50μM DP
NB
NB+100μM FeCl3

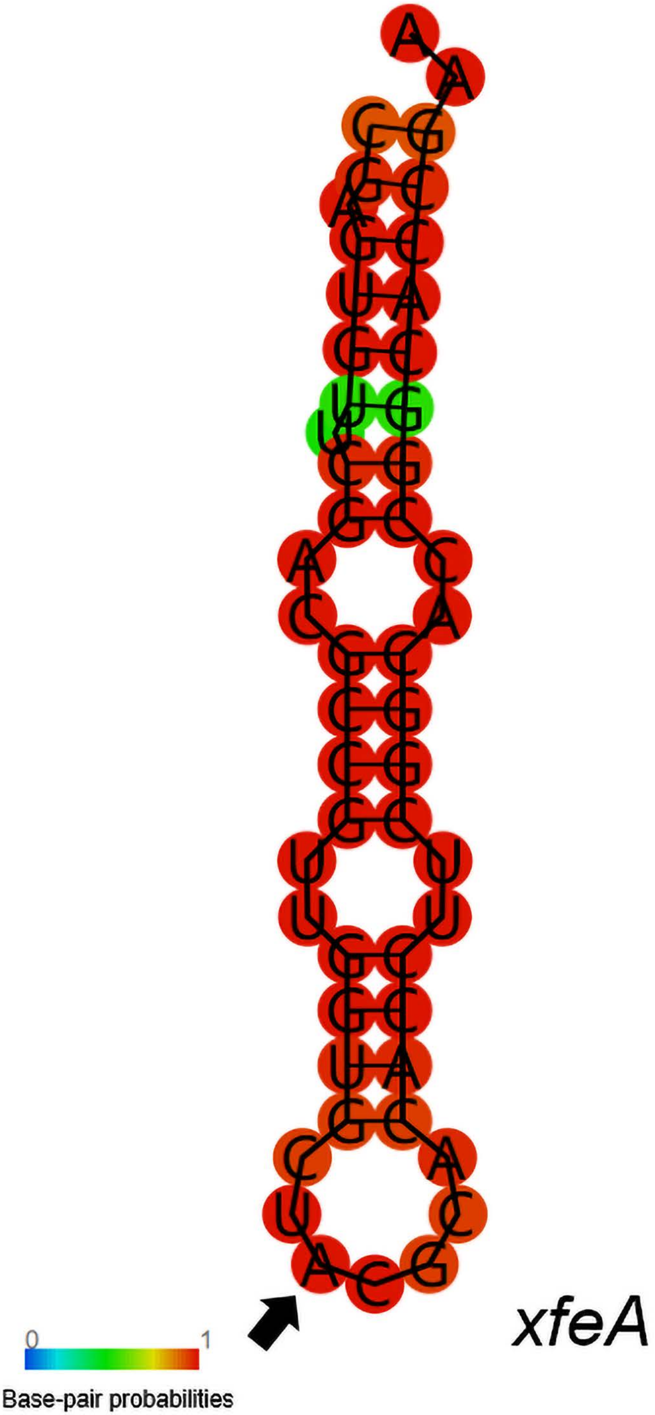
xfeA
0
1
Base-pair probabilities

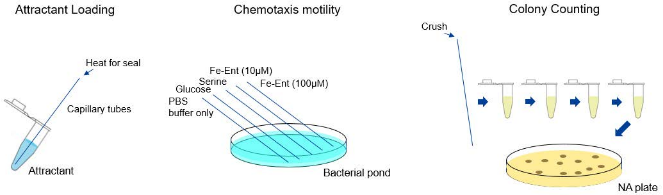
Attractant Loading
Heat for seal
Capillary tubes
Attractant
Chemotaxis motility
Fe-Ent (10μM)
Serine
Fe-Ent (100μM)
Glucose
PBS
buffer only
Bacterial pond
Colony Counting
Crush
NA plate

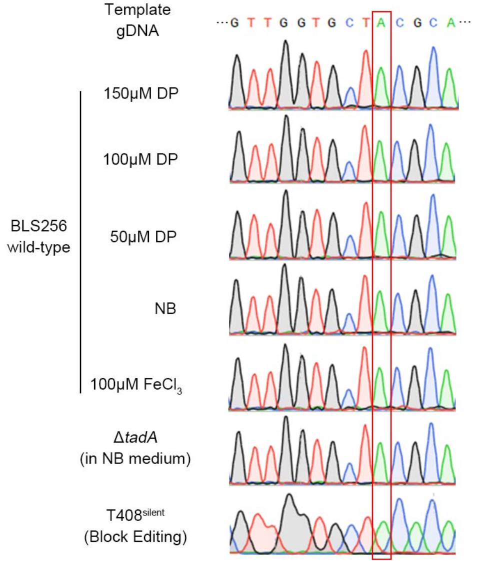

Template gDNA
···G T T G G T G C T A C G C A···
BLS256 wild-type
150μM DP
100μM DP
50μM DP
NB
100μM FeCl3
ΔtadA (in NB medium)
T408silent (Block Editing)

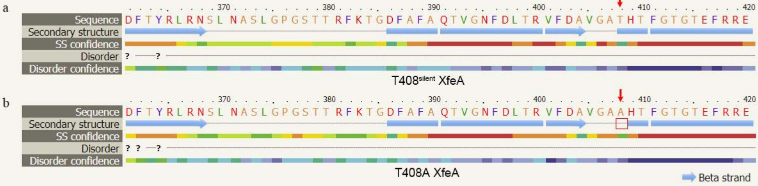
a
Sequence
Secondary structure
SS confidence
Disorder
Disorder confidence
370
380
390
400
410
420
DFTYRLRNSLNASLGPGSTTRFKTGDFAFAQTVGNFDLTRVFDAVGATHTFGTGTEFRRE
T408silent XfeA
b
Sequence
Secondary structure
SS confidence
Disorder
Disorder confidence
DFTYRLRNSLNASLGPGSTTRFKTGDFAFAQTVGNFDLTRVFDAVGAAHTFGTGTEFRRE
T408A XfeA
Beta strand

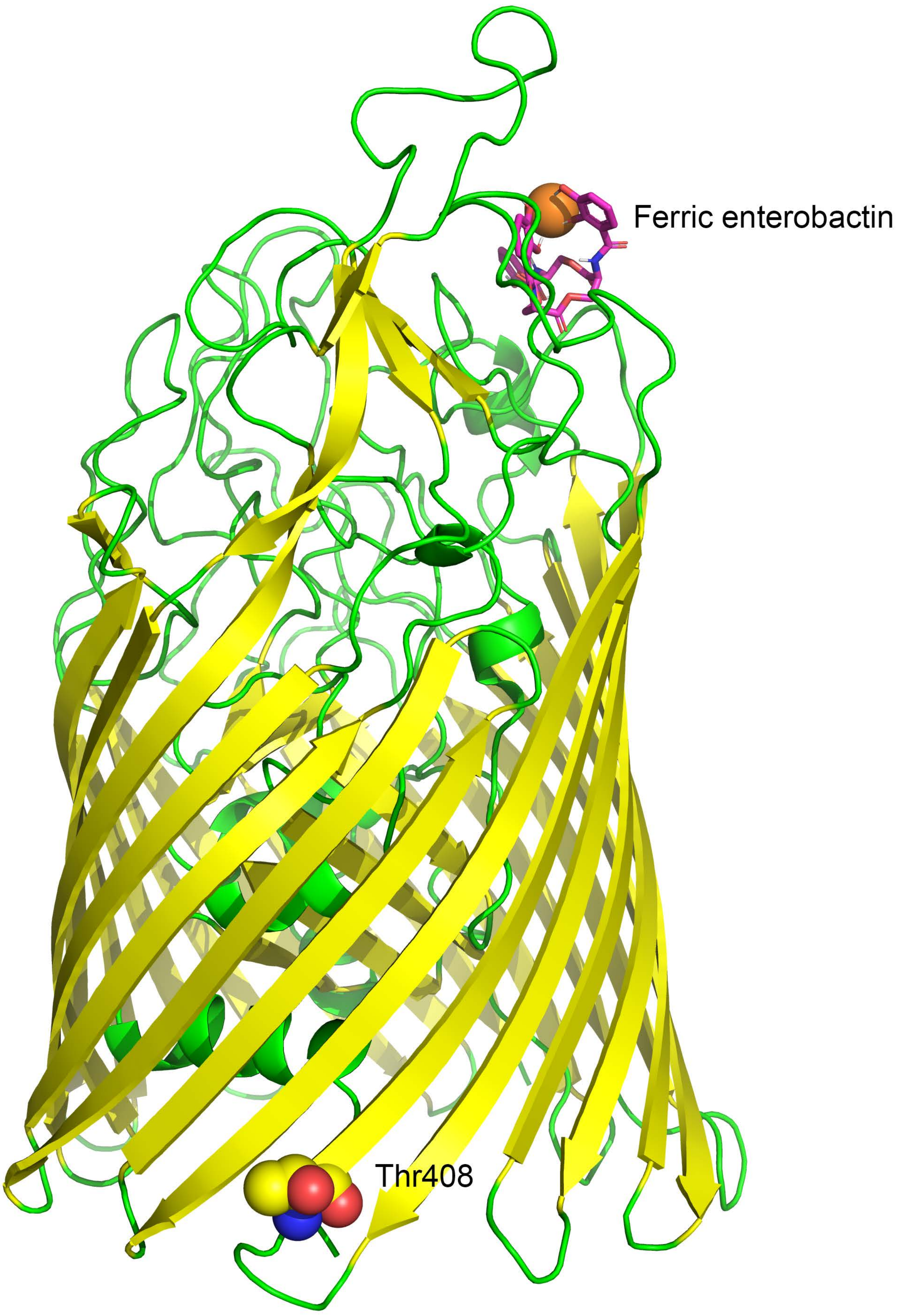

Ferric enterobactin
Thr408

Table S1 Strains and plasmids used in this study.

Strain or plasmid	Relevant characteristics	Source or reference
Strains		
Escherichia coli		
DH5α	F^- Φ80d *lac*ZΔM15Δ(lacZYA-argF) U169 *recA1 endA1, hsdR17*(r_k^-,m_k^+) *phoA supE44*λ^- *thi-1 gyrA96 relA1*	
BL21(DE3)	F^- *ompT hsdS*(r_B^- m_B^-) *gal dcm* (DE3)	Transgen Biotech, Beijing, China
BL21-xoc2282	BL21(DE3) harboring pET-30a::*xoc_2282*; Km^r	This study
BL21-xoc2291	BL21(DE3) harboring pET-30a::*xoc_2291*; Km^r	This study
Xanthomonas oryzae pv. *oryzicola*		
BLS256	Wild-type	(Bogdanove et al., 2011)
T408A	BLS256 containing a mutation (ACG to GCG) that changes Thr to Ala at residue 408 in XfeA	This study
$T408^{silent}$	BLS256 containing a synonymous mutation (ACG to ACA) in *xfeA* that blocks A-to-I RNA editing at amino acid 408	This study
Plasmids		
pKMS1	Km^R; R6K-based suicide vector; requires the *pir*-encoded π protein for replication	(Li et al., 2011)
pET-30a(+)	Km^R; contains N-terminal His-/thrombin/S-tag/enterokinase and C-terminal His-tag sequence; expression controlled by T7 RNA polymerase	Novagen, Madison, WI, USA
pKMS1::xfeA	Fragments encoding *xfeA* were amplified from *Xoc* BLS256 with primers *xfeA* F/R (Table S2), digested with *Sal*I/*Xho*I, and subcloned into the *Sal*I/*Xho*I site of pKMS1	This study
pKMS1::T408A	Contains A to G point mutation in *xfeA* that changes amino acid residue 408 from Thr to Ala; cloned in pKMS1 for homologous recombination, Km^R	This study
pKMS1::$T408^{silent}$	Contains G to A point mutation in *xfeA* that blocks A-to-I RNA editing in residue 408; cloned in pKMS1 for homologous recombination, Km^R	This study
pET-30a::*xoc_2282*	Contains a 2382 bp *Bam*HI/*Hin*dIII fragment encoding *xoc_2282* in pET-30a(+), Km^R	This study

pET-30a::*xoc_2291*	Contains a 2109 bp *Bam*HI/*Hin*dIII fragment encoding *xoc_2291* in pET-30a(+), Km^R	This study

[a] Km^R, kanamycin resistance.

References

Bogdanove, A.J., Koebnik, R., Lu, H., Furutani, A., Angiuoli, S.V., Patil, P.B., Van Sluys, M.-A., Ryan, R.P., Meyer, D.F., and Han, S.-W. (2011). Two new complete genome sequences offer insight into host and tissue specificity of plant pathogenic Xanthomonas spp. J Bacteriol *193*, 5450-5464.

Li, Y.-R., Zou, H.-S., Che, Y.-Z., Cui, Y.-P., Guo, W., Zou, L.-F., Chatterjee, S., Biddle, E.M., Yang, C.-H., and Chen, G.-Y. (2011). A novel regulatory role of HrpD6 in regulating hrp-hrc-hpa genes in *Xanthomonas oryzae* pv. *oryzicola*. Mol Plant Microbe Interact *24*, 1086-1101.

Table S2. Primers used in this study

Application		Primers[a]	Sequences (5' to 3')[a,b]
T408A point mutation	Amplification of *xfeA*	*xfeA* F (Sa)	GCGTCGACACAGCAAGATCGGCAAGG
		xfeA R (Xh)	CCCTCGAGGGTAGTTGTCCAGGATGTTCT
	Generation of T408A point mutation in *xfeA*	T408A F	TCGACGCCGTTGGTGCTGCGCACACCTTCG
		T408A R	CAGCACCAACGGCGTCGAACACTCGGGTCA
T408[silent]	Generation of T408 point synonymous mutation in *xfeA*	T408Silent F	GACGCCGTTGGTGCTACACACACCTTCGGCA
		T408Silent R	TGTAGCACCAACGGCGTCGAACACTCGGGT
Amplification of *xfeA* cDNA including T408 point mutation		c T408 F	CGCAAACCGTGGGCAATT
		c T408 R	CCAGATCAGTGGAGAACTTGTC
Amplification of *xoc_2282*		*xoc_2282* F (H)	CCAAGCTTGAACTCTTGCCAGTGACCGTC
		xoc_2282 R (B)	CGGGATCCATGCAATGGATCAACAATCTG
Amplification of *xoc_2291*		*xoc_2291* F (H)	CCAAGCTTGAACTCCTGCCAGCTGGTC
		xoc_2291 R (B)	CGGGATCCATGAACGACCATACCTATCAG
qPCR analysis of *xfeA*		q *xfeA* F	CCTGGCTCAACACCAAGATT
		q *xfeA* R	GCATCGTAATCCCAGTTGCC
qPCR analysis of *xoc_2278*		q *xoc_2278* F	CGATGGCGATCAGACGAT
		q *xoc_2278* R	GTCAGCAAGGTCAAGATGG

qPCR analysis of *xoc_2280*	q *xoc_2280* F	ATCACATTGCGACAGAACA
	q *xoc_2280* R	CAAGTGCCGAGTCATTCC
qPCR analysis of *xoc_2289*	q *xoc_2289* F	GTAGATCAGCGTGGACAC
	q *xoc_2289* R	CTTGAGCGAACAGACCTT
qPCR analysis of *xoc_2291*	q *xoc_2291* F	GAGGCTTTCCACCATCAC
	q *xoc_2291* R	CCTGTCTTCGGTCAATCG
qPCR analysis of *xoc_2297*	q *xoc_2297* F	ATCTGCTCTGCTTGGTAG
	q *xoc_2297* R	GCGTATCGTCTGATATTCG
qPCR analysis of *rpoD* (internal control)	*rpoD* F	CGACAACACCACCAACATCAATC
	rpoD R	GCTTACCGACCTCTTCCAACG

[a] The following restriction sites were introduced into primer sequences: B, *Bam*HI; H, *Hind*III; Sa, *Sal*I; and Xh, *Xho*I. Restriction sites are underscored in the primer sequences.

[b] Nucleotides in red font indicate introduction of a point mutation.
